# Vaccine Hesitancy of COVID-19 among Parents for Their Children in Middle Eastern Countries—A Systematic Review

**DOI:** 10.3390/vaccines11101556

**Published:** 2023-10-01

**Authors:** Muhammad Shahid Iqbal, Salah-Ud-Din Khan, Shafqat Qamer, Saeed Vohra

**Affiliations:** 1 Department of Clinical Pharmacy, College of Pharmacy, Prince Sattam bin Abdulaziz University, Al-kharj 11942, Saudi Arabia; 2 Department of Biochemistry, College of Medicine, Imam Mohammad Ibn Saud Islamic University (IMSIU), Riyadh 11432, Saudi Arabia; 3 Department of Basic Medical Sciences, College of Medicine, Prince Sattam bin Abdulaziz University, Al-kharj 11942, Saudi Arabia; 4 Department of Anatomy, College of Medicine, Imam Mohammad Ibn Saud Islamic University (IMSIU), Riyadh 11432, Saudi Arabia

**Keywords:** COVID-19 vaccine, Middle Eastern countries, vaccine hesitancy, children

## Abstract

The current systematic review presents COVID-19 vaccine hesitancy among parents for their children in Middle Eastern countries. Moreover, the vaccine acceptance rate of parents from the Middle East and the factors effecting the acceptance rate were reviewed and summarized. For this systematic review, basic electronic academic databases (Scopus, Science Direct, ProQuest, Web of Science and PubMed) were used for the search, along with a manual search on Google Scholar. This systematic review was conducted by following the “Preferred Reporting Items for Systematic Reviews and Meta-Analyses (PRISMA)” guidelines. Moreover, utilizing the framework of the PECO-S (Population Exposure Comparison Outcome Study design), various observational studies were recruited for this review. Out of 2123 studies, 25 studies meeting the inclusion criteria were included in the current review. All of the included studies were about parental vaccine hesitancy for COVID-19 in Middle Eastern countries and published during 2020–2022. Overall, 25 research papers comprising 10 different Middle Eastern countries with 33,558 parents were included. The average age of parents was 39.13 (range: 18–70) years, while the mean age of children was 7.95 (range: 0–18) years. The overall hesitancy rate was 44.2% with a SD of ± 19.7. The included studies presented enhanced COVID-19 vaccine hesitancy among parents in Middle Eastern countries. The lower vaccine acceptance rate among parents was mainly because of a fear of the potential side effects. Furthermore, the lack of information regarding vaccine safety and efficacy, the fear of unreported side effects and concerns about the authenticity of vaccine development and preparation were the predictors of parental COVID-19 vaccine hesitancy among Middle Eastern countries.

## 1. Introduction

The SARS-CoV-2 “severe acute respiratory syndrome coronavirus” is the causative agent of an infectious disease called COVID-19. This devastating respiratory tract infection was initially observed in “Wuhan”, the capital of the province Hubei, China, in December of 2019, which then swiftly spread worldwide [1,2,3,4]. The World Health Organization (WHO) officially declared COVID-19 a pandemic in March of 2020 [2,3,4]. The COVID-19 pandemic resulted in vicious morbidity and mortality worldwide and shattered global economies [3]. According to the WHO dashboard of COVID-19, the officially reported confirmed cases of COVID-19 infection as of 20 May 2022 were 521,920,560, with 6,274,323 reported deaths worldwide [4]. Furthermore, nearly 7.98 billion, i.e., 65.78% of the world’s population had received their first dose of the COVID-19 vaccination by May 2022 [3]. Hence, till July 2022, the global strategy of the WHO regarding “complete COVID-19 vaccination” was only halfway accomplished, especially in low-income and developing countries [5].

Since medieval times, vaccine hesitancy and rejection has been a pressing issue, especially in developing countries, including Middle Eastern countries [1,2,3,4]. Moreover, in the past few decades, the incidences of vaccine rejection have also increased worldwide [6]. Furthermore, due to a high vaccine rejection rate, the prevalence of vaccine-preventable diseases has also been significantly increased in recent years [2]. In fact, parents’ or guardians’ consent is required for the vaccination of children under the age of 18 years. Therefore, parents’ attitude, beliefs and behavior towards vaccine acceptance always play a significant role in children’s vaccination [7,8]. Parental vaccine hesitancy, despite the availability of the vaccines, is a prime barrier in childhood immunization [5].

Undeniably, COVID-19 was a rare infection that significantly contributed to plenteous causalities worldwide. However, the COVID-19 vaccine was developed and synthesized in the least time possible [9]. Therefore, the general population all over the world, especially parents, had concerns regarding the authenticity, developmental protocols and validation procedures of the newly developed COVID-19 vaccines [10,11]. Hence, this review was designed to observe and present the parental hesitancy/rejection rate, and acceptance rate of COVID-19 vaccines in vaccinating their children against COVID-19. Specifically, the present systematic review focuses on parents’ attitude towards vaccinating their children against COVID-19 across Middle Eastern countries. Moreover, the current systematic review will also identify the predictors and factors related to the hesitant attitude of parents towards the COVID-19 vaccination. The observations of this review will be helpful in understanding various parental concerns regarding child COVID-19 vaccines. This systematic review will also provide a basis for designing and implementing targeted healthcare strategies by concerned authorities, specifically in Middle Eastern countries.

## 2. Materials and Methods

### 2.1. Study Design

The Cochrane handbook was used as the main source of guidance for this systematic review. Moreover, the study protocols were in accordance with the PRISMA flow statement guidelines. The keywords used for finding the research studies included ‘COVID-19 vaccine’, ‘children COVID-19 vaccination’, ‘COVID-19 vaccination in Middle Eastern countries’, ‘parental COVID-19 vaccine hesitancy’ and ‘parental acceptance rate of COVID-19 vaccine’. For the present systematic review, electronic databases (Scopus, Science Direct, ProQuest, Web of Science and PubMed) were used for the study search, along with a manual search on Google Scholar. Utilizing the framework of the PECO-S (Population Exposure Comparison Outcome Study design), various observational studies were recruited. The search was restricted to the English language and COVID-19 vaccine studies published from January 2020 to December 2022 were included. Out of 2123 studies obtained, 25 studies that met the inclusion criteria were included in this systematic review.

### 2.2. Inclusion Criteria

The inclusion criteria were as follows:

(1) The study population were parents.
(2) The studies were from Middle Eastern countries.
(3) The studies were on the COVID-19 vaccine for children.
(4) The study design of the included studies was observational cross-sectional.
(5) The studies were published in the English language.
(6) The studies were published in peer-reviewed journals.

### 2.3. Exclusion Criteria

The exclusion criteria were as follows:

(1) The studies with general population or healthcare providers.
(2) The studies outside of Middle Eastern countries.
(3) The studies with study design other than observational cross-sectional.
(4) The studies published in languages other than English.

### 2.4. Data Extraction

The data extracted from the included studies were comprised author details, year of the study, country of the study, mean age and educational background of the parents in the included studies, study design, sample size, parental vaccine acceptance rate, parental vaccine hesitancy rate and predictors (reported reasons) of the vaccine hesitancy among the parents. The “Cochrane Bias” tool of LvE for risk assessment was utilized to rule out the risk of biasness. This biasness assessment was further verified by KT, HdG and LvD.

### 2.5. Data Synthesis and Analysis

The parental COVID-19 vaccine acceptance rate was accessed in all of the included studies. Moreover, the parental COVID-19 vaccine hesitancy rate as well as the predictors (reasons) of COVID-19 vaccine hesitancy were also investigated in all of the included studies.

## 3. Results

**Figure 1:**
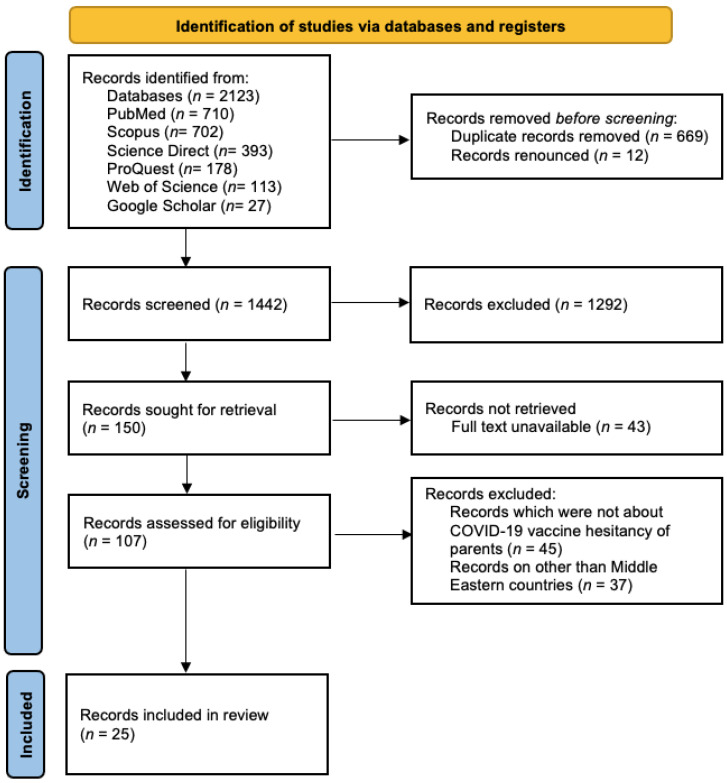
PRISMA flow diagram.

Through an electronic database search, a total of 2123 original research articles were identified, from which 1442 studies were screened after removing the duplicate records and renounced records. Among the 1442 screened studies, 1292 records were excluded that did not match the required keywords, and 150 study reports were shortlisted. Among the 150 shortlisted research articles, 43 studies were found as conference proceedings (abstract) only when the full-text articles were searched for. They were published in the special edition/issue of the journals as abstracts and were not available as full texts. After excluding them, a total of 107 studies were left for further evaluation. And among the 107 shortlisted studies, 45 studies were excluded as they were not about parental hesitancy towards the COVID-19 vaccine. Afterwards, a total of 37 studies that were conducted in countries other than Middle Eastern countries were also excluded. At the end, 25 studies were screened that met the inclusion criteria and were evaluated for the present systematic review. The details are described in the PRISMA flow diagram (Figure 1).

For assessing the quality of cross-sectional studies, many tools are available, such as the Newcastle–Ottawa scale (NOS) adapted for cross-sectional studies, the NIH quality assessment tool (NIH-QAT) for observational cohort and cross-sectional studies, the Johanna Briggs institute checklist (JBIC) for analytical cross-sectional studies, and the appraisal tool for cross-sectional studies (AXIS tool/AXIS 20). We used the points AXIS 20 tool to check the quality of all the included studies. The AXIS tool is a critical appraisal tool that addresses issues in cross-sectional studies and reports on quality as well as the risk of bias in cross-sectional studies. The AXIS tool (developed in 2016), also known as AXIS 20, is a 20-point questionnaire that addresses key areas in cross-sectional studies, i.e., study design, sample size with justification, target population, sampling technique, validity and reliability of the study, and overall methodology of the study [12]. Table 1 presents the results of the quality assessment of the included studies using AXIS 20. The aims and objectives were clearly defined in the included studies. The majority of the included studies had an adequate study design and appropriate sample size. The results of the included studies were clearly defined and consistent. Moreover, no conflicts of interest were observed in any study that was included in this systematic review.

The study settings (country of research), data collection time interval, study design, sample size, parental acceptance rate, hesitancy/rejection rate, as well as the predictors (reasons) for parental hesitancy were analyzed. All of the studies recruited for this review were observational, cross-sectional studies. Among all of the 25 included studies, only 1 study was published in 2020, 22 studies were published in 2021, and only 2 studies were published in 2022. The characteristics of the included studies are presented in Table 2.

Among the added research studies from Middle Eastern countries, the majority of the studies had clear objectives. On the other hand, none of the included studies had any funding sources or conflicts of interest that may affect the authors’ interpretation of the results. The majority of the included studies had a clearly defined target/reference population, except the study conducted by Sara et al. [15]. Some studies, such as those of Elkhadry SW et al. [16], Yulia G et al. [18], and Özlem A et al. [21], did not have a justified sample size. Similarly, all the included studies had ethical approval and the consent of the study participants.

Among the recruited studies from Middle Eastern countries, the majority of the studies, i.e., 11 original research studies, were from Saudi Arabia (KSA), 7 original research studies were from Turkey, 3 studies were from Jordan and 3 studies were from Israel. The rest of the studies included in this systematic review were from Qatar, Egypt, Iraq, Kuwait, Lebanon, Palestine and the United Arab Emirates (UAE). All of the added studies focused on the parents’ hesitancy regarding the COVID-19 vaccination for their children. The study duration for most of the studies was about 2–3 months.

## 4. Discussion

After the global outbreak of COVID-19, the main focus of healthcare authorities was to develop herd immunity among individuals worldwide [7]. Due to the highly infectious nature of COVID-19, it became necessary to immunize the maximum possible percentage of the population to minimize its widespread transmission [3]. Vaccine hesitancy and refusal had been a major issue faced by healthcare authorities across the globe [32]. Parental hesitancy regarding childhood immunization was a crucial problem that had affected a substantial number of children worldwide [36]. Multiple studies have been conducted regarding COVID-19 vaccine acceptance and the hesitancy towards it among parents in different parts of the world [9]. Similarly, several studies have also been conducted on the parental acceptance and hesitancy of routine vaccines [36]. Interestingly, it was observed that parents’ attitudes towards routine childhood immunization were more positive and accepting than towards COVID-19 vaccines, and they were more hesitant towards the COVID-19 vaccination for their children [35]. The present systematic review is focused on the parental COVID-19 vaccine acceptance rate, hesitancy or refusal rate, along with factors (predictors) affecting parental COVID-19 vaccine hesitancy across Middle Eastern countries.

The general findings of this review present a clear picture: that the majority of parents in Middle Eastern countries were hesitant to get their children vaccinated against COVID-19. Similarly, a study conducted in Bangladesh presented a high percentage of parental vaccine hesitancy regarding the COVID-19 vaccination for their children [11]. However, these findings are contrary to the findings of studies conducted across various European countries, where the parental COVID-19 vaccine acceptance rate for their children was much higher [36]. Similarly, the COVID-19 vaccine acceptance rate of Italian parents for their children was higher as compared to in Arab countries [37]. The percentage of the population that has been fully vaccinated against COVID-19 in the USA, Italy, Hong Kong and UAE were at 36%, 13.7%, 10% and 38.8% as of May 2021, respectively [36]. Moreover, an observational cross-sectional study conducted on an American population also reported similar results, i.e., a high parental acceptance rate for the COVID-19 vaccine and a low hesitancy or refusal rate as compared to Middle Eastern countries [38].

Furthermore, some other studies presented somewhat similar results to the US but opposite to Middle Eastern countries, i.e., parents’ attitudes towards the COVID-19 vaccine for their children were more accepting as compared to Middle Eastern countries [7,9,10,37]. Likewise, a cross-sectional study conducted across the United Kingdom also presented a clear picture of the vaccine hesitancy rate among parents, but the parental COVID-19 vaccine hesitancy was still comparatively low as compared to Middle Eastern parents, especially Arabian mothers [30].

The present review depicts multiple reasons associated with parental COVID-19 vaccine hesitancy for their children across Middle Eastern countries. The most common reason for parental COVID-19 vaccine refusal and hesitancy was a lack of adequate information about the side effects of the COVID-19 vaccines. Another reason was the fear of acquiring COVID-19 infections even after the COVID-19 vaccination. It can be said that parents in Middle Eastern countries had trust issues regarding the COVID-19 vaccine synthesis and its development. Primarily, the COVID-19 vaccines were synthesized and prepared in a very short time, which raised a sense of mistrust among Middle Eastern parents. This in turn resulted in greater COVID-19 vaccine hesitancy and an increased refusal rate among parents for their children.

Insufficient information regarding the COVID-19 vaccine efficacy and safety was the second most commonly reported reason among the parents who participated in the included studies. An absence of reliable scientific information regarding the COVID-19 vaccine effectiveness also resulted in high COVID-19 vaccine hesitancy and reluctance among Middle Eastern parents. These results are better but contrary to the studies conducted in Asian countries such as India, Pakistan and Bangladesh, where parents presented COVID-19 vaccine hesitancy for themselves as well as for their children due to fake news, falsified myths, religious beliefs and self-imagined phony ideologies [39].

The findings of the present systematic review, in terms of the safety of the COVID-19 vaccines, are similar to the findings of the studies conducted in different European countries, where the main reason for parental COVID-19 vaccine hesitancy for children was the fear of unwanted side effects, poor COVID-19 vaccine safety knowledge and a lack of vaccine safety literature availability for the general population [6]. Likewise, most parents across the United States, as well as the United Kingdom, reported fear of the COVID-19 vaccine side effects as a major predictor of COVID-19 vaccine hesitant behavior [3,10,11,40].

The parental hesitancy rate for COVID-19 vaccines was observed to be 44.2% with a SD of ± 19.7% across Middle Eastern countries. Similarly, a cross-sectional study conducted on 3009 parents in eastern China presented similar results. According to that study, parental hesitancy regarding child routine immunization and COVID-19 immunization was around 40.7% [41]. Moreover, another study conducted among 223 parents presented an overall parental hesitancy of 99.4% regarding routine childhood immunizations [42]. In contrast, a cross-sectional study from Italy (437 Italian parents) reported less hesitancy regarding routine childhood immunizations. The Italian study demonstrated 34.7% parental hesitancy regarding routine childhood immunizations [43]. Similarly, another exploratory study among 199 parents from the United States presented 24% hesitancy regarding the influenza vaccine for their children [44].

The findings of the present systematic review, which are based on various studies across Middle Eastern countries, present a crucial need for healthcare authorities and health policy makers to address the reasons for COVID-19 vaccine hesitancy among parents. Furthermore, healthcare authorities should also promote productive, beneficial and valuable awareness regarding the benefits of the COVID-19 vaccine for children. Parents and guardians should also be instructed thoroughly about the long-term consequences of the COVID-19 vaccine. The parental COVID-19 vaccine acceptance rate would be enhanced if parents were well-informed and aptly guided about the potential benefits of the COVID-19 vaccines.

### Strength and Limitations

The current systematic review, which is based on studies conducted in different Middle Eastern countries, presents an enormous perspective on parental COVID-19 vaccine acceptance and hesitancy. The included studies had large sample sizes. Therefore, this review brings to light the vaccination attitude of a large number of parents from Middle Eastern countries. The studies excluded from this review were in languages other than English or were conference proceedings as abstracts that were not available as full-length papers. Excluding such studies from this systematic review represents a small hindrance in understanding the parental trends and attitude towards COVID-19 vaccination for children. Additionally, this review did not recruit other subgroups of the professional population (healthcare professionals and workers) or the general population.

Further work should be conducted to explore the COVID-19 vaccine acceptance and hesitancy rates and the reasons for this hesitancy among the general population and other professional subgroups of the population. Such data could be utilized by the local healthcare authorities to identify vaccination barriers and enhance vaccine acceptance.

## 5. Conclusions

The findings of this review revealed that most parents from Middle Eastern countries were hesitant to get their children vaccinated against COVID-19. The majority of the participants from the studies recruited for this systematic review were mothers. A few major reasons for the low acceptance rate and high hesitancy of the COVID-19 vaccines among parents were a lack of information regarding vaccine safety and efficacy and a fear of unreported side effects and concerns about the authenticity of the vaccine development and preparation. The findings from the current systematic review could be used by healthcare policy makers to design, compose and implement strategies that target and address COVID-19 vaccination barriers among parents.

## Figures and Tables

**Table 1:** Quality assessment of included studies using AXIS tool.

	Study 1 [13]	Study 2 [14]	Study 3 [15]	Study 4 [16]	Study 5 [17]	Study 6 [18]	Study 7 [19]	Study 8 [20]	Study 9 [21]	Study 10 [22]	Study 11 [23]	Study 12 [24]	Study 13 [8]	Study 14 [25]	Study 15 [26]	Study 16 [7]	Study 17 [27]	Study 18 [28]	Study 19 [29]	Study 20 [30]	Study 21 [31]	Study 22 [32]	Study 23 [33]	Study 24 [34]	Study 25 [35]
**Introduction**																									
Were the aims/objectives of the study clear?	✔	✔	✔	✔	✔	✔	✔	✔	✔	✔	✔	✔	✔	✔	✔	✔	✔	✔	✔	✔	✔	✔	✔	✔	✔
**Methods**																									
Was the study design appropriate for the stated aim(s)?	✔	✔	✖	✔	✔	✔	✔	✔	✔	✖	✔	✖	✔	✔	✔	✔	✔	✔	✔	✔	✔	✔	✔	✔	✔
Was the sample size justified?	✔	✔	✔	✖	✔	✖	✔	✔	✖	✔	✔	✔	✔	✔	✔	✔	✔	✔	✔	✔	✔	✔	✔	✔	✔
Was the target/reference population clearly defined?	✔	✔	✖	✔	✔	✔	✔	✔	✔	✔	✔	✔	✔	✔	✔	✔	✔	✔	✔	✔	✔	✔	✔	✔	✔
Was the sample frame taken from an appropriate population base so that it closely represented the target/reference population under investigation?	✔	✔	✔	✔	✖	✔	✔	✖	✖	✔	✖	✔	✔	✔	✔	✖	✔	✔	✖	✔	✔	✔	✔	✖	✖
Was the selection process likely to select subjects/participants that were representative of the target/reference population under investigation?	✔	✔	✖	✔	✔	✖	✔	✔	✔	✖	✔	✔	✔	✔	✔	✖	✔	✔	✖	✔	✔	✔	✔	✖	✖
Were measures undertaken to address and categorize non-responders?	✔	✔	✔	✖	✔	✔	✖	✔	✔	✔	✖	✖	✔	✖	✖	✔	✔	✔	✖	✖	✔	✔	✖	✖	✔
Were the risk factor and outcome variables measured appropriate to the aims of the study?	✔	✔	✔	✖	✔	✔	✔	✔	✔	✔	✔	✔	✔	✔	✖	✔	✔	✔	✔	✔	✔	✔	✔	✔	✔
Were the risk factor and outcome variables measured correctly using instruments/measurements that had been trialed, piloted or published previously?	✔	✔	✔	✖	✔	✔	✔	✖	✔	✔	✔	✔	✔	✖	✔	✔	✔	✔	✖	✔	✔	✔	✔	✔	✔
Was it clear what was used to determine statistical significance and/or precision estimates? (e.g., *p*-values, confidence intervals)	✔	✔	✔	✔	✔	✖	✔	✔	✔	✔	✔	✔	✔	✔	✔	✔	✔	✔	✔	✔	✔	✔	✔	✔	✖
Were the methods (including statistical methods) sufficiently described to enable them to be repeated?	✔	✔	✔	✔	✔	✖	✔	✔	✔	✔	✔	✔	✔	✔	✔	✔	✔	✔	✔	✔	✔	✔	✔	✔	✖
**Results**																									
Were the basic data adequately described?	✔	✔	✔	✔	✔	✔	✔	✔	✔	✔	✔	✔	✔	✔	✔	✔	✔	✔	✔	✖	✔	✔	✖	✔	✖
Does the response rate raise concerns about non-response bias?	✖	✖	✖	✖	✖	✖	✖	✖	✖	✖	✖	✖	✖	✖	✖	✖	✖	✖	✖	✖	✖	✖	✖	✖	✔
If appropriate, was information about non-responders described?	✖	✖	✖	✖	✔	✖	✖	✔	✔	✔	✖	✖	✖	✖	✖	✔	✖	✖	✖	✖	✖	✖	✔	✖	✔
Were the results internally consistent?	✔	✔	✔	✔	✔	✔	✔	✔	✔	✔	✔	✔	✔	✔	✔	✔	✔	✔	✔	✖	✔	✔	✖	✔	✖
Were the results presented for all the analyses described in the methods?	✔	✔	✔	✔	✔	✔	✔	✔	✔	✔	✔	✔	✔	✔	✔	✔	✔	✔	✔	✖	✔	✔	✖	✔	✖
**Discussion**																									
Were the authors’ discussions and conclusions justified by the results?	✔	✔	✔	✔	✔	✔	✔	✔	✔	✔	✔	✔	✔	✔	✔	✔	✔	✔	✔	✖	✔	✔	✖	✔	✖
Were the limitations of the study discussed?	✔	✔	✔	✔	✔	✔	✔	✔	✔	✔	✔	✔	✔	✔	✔	✔	✔	✔	✔	✖	✔	✔	✖	✔	✖
**Other**																									
Were there any funding sources or conflicts of interest that may affect the authors’ interpretation of the results?	✖	✖	✖	✖	✖	✖	✖	✖	✖	✖	✖	✖	✖	✖	✖	✖	✖	✖	✖	✖	✖	✖	✖	✖	✖
Was ethical approval or consent of participants attained?	✔	✔	✔	✔	✔	✔	✔	✔	✔	✔	✔	✔	✔	✔	✔	✔	✔	✔	✔	✔	✔	✔	✔	✔	✔

✔ = Yes, ✖ = No

**Table 2:** Study characteristics of the included studies.

No.	Study	Country	Mean Age and Education Level of the Parents	Study Design	Sample Size (N)	Acceptance Rate (%)	Hesitancy/Rejection Rate (%)	Reason for Hesitancy (Determinants of Vaccine Hesitancy)
1.	Yigit, M. et al., 2021 [13]	Turkey	Mean age = 39.7 College level 61.9%	Cross-sectional Non-random sampling Face-to-face interviews	428 parents 63.6% mothers	The overall vaccine acceptance rate of parents was 34.4%.	66.1% of parents (majorly mothers) were hesitant to receive foreign vaccine.	Lack of knowledge regarding side effects of vaccine or not trusting the vaccines being imported from abroad/foreign countries.
2.	Temsah M-H, Alhuzaimi AN et al., 2021 [14]	Saudi Arabia	Mean age = 36 years College level 88.9%	Cross-sectional Non-random sampling Web-based survey	3167 parents	The vaccine acceptance rate was 52.4% in their study.	47.6% refused to vaccinate their children against COVID-19.	The most common reason for vaccine hesitancy was insufficient information and side effect worries.
3.	Sarah Musa et al., 2021 [15]	Qatar	Not Given	Retrospective Cross-sectional Non-random sampling Online survey	4023 parents	Among study subjects, 82.10% parents accepted the vaccine for their children.	Vaccine hesitancy rate was 17.9%, such low rate of vaccine hesitancy could be attributed to health awareness campaigns.	Age groups, manufacturer/country, and previous COVID-19 recoveries were the predictors of vaccine hesitancy.
4.	Elkhadry et al., 2022 [16]	Egypt	Mean age = 38.8 years Illiterate 92%	Cross-sectional Random sampling Online survey	173 parents	Among 173 parents, only 18.5% were willing to vaccinate their child against COVID-19.	81.5% of parents presented vaccine hesitancy for their children. Mothers were more hesitant (68.2%) as compared to fathers (31.8%).	Socio-economic status and education level of parents were the predictors of vaccine hesitancy. Parents presented trust issues with vaccine due to inadequate information.
5.	Meltem Yılmaz et al., [17]	Turkey	Mean age = 35 years College level 82.8%	Cross-sectional Web-based survey	1035 parents	Vaccine acceptance rate for their children was 36.3% among Turkish parents. The enhanced vaccine acceptance was observed in parents who were healthcare providers.	Around 63.7% of parents were hesitant regarding COVID-19 vaccine for their children.	Willingness of parents and positive attitude of parents towards vaccine were the predictors for enhanced vaccine acceptance.
6.	Yulia Gendler and Lani Ofri 2021 [18]	Israel	Mean age = 44.7 years College level 73.7%	Cross-sectional Web-based survey	520 parents	The vaccine acceptance rate was 70.4% among Israeli parents. The higher acceptance rates could be attributed to the information provided through healthcare providers and internet sources.	A total of 29.6% of parents were hesitant to vaccinate their children against COVID-19.	Parent’s literacy regarding vaccines and the positive perception and attitude towards vaccines were the predictors for vaccine acceptance.
7.	Moawiah Khatatbeh et al., 2022 [19]	Iraq Jordan Kuwait Lebanon Palestine Saudi Arabia UAE	Mean age = 37 years College level 85.5%	Cross-sectional Descriptive Non-random sampling Web-based survey	3744 parents (from 8 countries)	Only 18.9% of the parents showed acceptance with covid-19 vaccine for their children.	81.1% of parents presented hesitancy towards COVID-19 vaccine and prevented their children from receiving the vaccine. Among which, 32.5% had trust issues regarding the safety and necessity of the COVID-19 vaccine.	Parent’s education, age, occupation and their own vaccine status were the predictors for vaccine hesitancy. The major reason for the hesitancy towards vaccines was a lack of trust and safety issues with the COVID-19 vaccine.
8.	Bader A. Altulaihi et al., 2021 [20]	Saudi Arabia	Mean age = 38 years College level 63%	Cross-sectional Web-based survey	333 parents	53.7% of the total parents recruited for this study presented willingness to vaccinate their children against COVID-19.	46.3% of parents were hesitant regarding COVID-19 vaccine.	Lack of adequate information and lack of evidence of safety of vaccine were the reasons for vaccine hesitancy among parents. Age of parents and history of previous vaccine acceptance attitude were the predictors of vaccine acceptance among parents.
9.	Özlem Akgün et al., 2022 [21]	Turkey	Mean age = 43 years College level 32%	Cross-sectional Web-based online survey	201 parents of children	Vaccine acceptance was 41.8% in Turkish parents. However, the vaccine acceptance rate in fathers and mothers were 64.2% and 57.7%, respectively.	The overall vaccine hesitancy rate was 45.8% among Turkish parents.	The major reason for the vaccine hesitancy and rejection was the fear of side-effects and any unknown interactions.
10.	Gonca Soyal et al., 2021 [22]	Turkey	Mean age = 22 years College level 46.4%	Descriptive study	1033 parents of 18–25 years old.	The COVID-19 vaccine acceptance rate observed through this study was 68.8%.	The COVID-19 vaccine hesitancy of parents was 11.4%. However, 3.1% of parents directly refused to accept vaccine for their children.	Two major reasons for vaccine hesitancy were presented by majority of the parents; Insufficient information regarding the COVID-19 vaccine benefits and absence of reliable information regarding harms of not getting COVID-19 vaccine.
11.	Soukaina Ennaceur et al., 2021 [23]	Saudi Arabia	Mean age = 33 years College level 67.8%	Cross-sectional Non-random sampling Online survey	379 parents of children <18 years of age	Overall, 167 parents (44%) gave acceptance of COVID-19 vaccine for their children.	A total of 212 parents (56%) were not willing to vaccinate their children. Among which, female parents were more hesitant as compared to male parents.	The main reason for vaccine hesitancy of lack of sufficient information about vaccine, fear of side effects and mistrust of healthcare system.
12.	Melike Y. Çelik., 2021 [24]	Turkey	Mean age = 34.1 years College level 69.3%	Descriptive study Snowball sampling technique	274 parents of children aged 0–12 years	Only 27.8% parents considered vaccinating their child with COVID-19 vaccine.	Overall, 128 parents (46.9%) were hesitant to get their children vaccinated against COVID-19, while 70 parents (25.5%) were undecided.	Majority of the parents were fearful regarding future side effects of vaccine. Moreover, some believed that vaccine had not gone through proper research.
13.	Altulahi, N. et al., 2021 [8]	Saudi Arabia	Mean age = 32 years College level 75.8%	Cross-sectional Non-random sampling Online survey	8056 parents of children <18 years of age.	Majority of the parents (51.67%) presented acceptance regarding COVID-19 vaccine for their children.	A total of 3893 parents (48.38%) presented hesitancy regarding COVID-19 vaccine for their children.	The major reason for refusal of COVID-19 vaccine was the lack of trust and belief in vaccine efficacy.
14.	Alsulaiman, J.W. et al., 2022 [25]	Jordan	Mean age = 35 years College level 89.3%	Cross-sectional Online survey	564 parents of 5–12 years of age children	Overall, the parents willing to get vaccinating of their children were 25.4%.	About 74.6% of parents were hesitant to get their children vaccinated against COVID-19.	Patients reported concerns regarding safety of the vaccine. Moreover, mistrust of health care system was the main reason for hesitancy to COVID-19 vaccine acceptance.
15.	Aldakhil, H. et al., 2021 [26]	Saudi Arabia	Mean age = 34 years College level 73.5%	Descriptive Cross-sectional Non-random sampling	270 female parents (mothers)	The acceptance rate of Saudi mothers regarding COVID-19 vaccine for their children was 24%, although 79% agreed on the importance of vaccine.	Only 24.31% presented hesitancy regarding child immunization against COVID-19.	The main reason for hesitancy regarding COVID-19 vaccine was the fear of side effects.
16.	Al-khlaiwi, T. et al., 2022 [7]	Saudi Arabia	Mean age = 44 years College level 86.6%	Cross-sectional Non-Random sampling Online survey	1304 parents With 26.2% male, 73.8% mothers	Among enrolled parents, 46.1% accepted the vaccine for their children.	Overall, 382 parents, i.e., 29.3%, presented hesitation to get their children vaccinated.	Children’s natural immune system alone is enough to fight coronavirus. That was the main reason for hesitancy.
17.	Çag, Y. et al., 2022 [27]	Turkey	Mean age = Not given College level (no %)	Cross-sectional Random sampling Interviews	1018 parents with 809 (79.5%) mothers	The acceptance rate of parents for their child vaccination against COVID-19 was 58.2%	Vaccine hesitancy rate was 21.7%. Whereas 20.1% parents refused to get their child vaccinated.	The reason for hesitant behavior was the fear of serious side effects as well as the lack of information.
18.	Atad, E. et al., 2021 [28]	Israel	Mean age = Not given College level (no %)	Cross-sectional Non-random sampling technique Online survey	1118 parents of children aged 12–15 years	Overall, 57.7% parents presented acceptance in getting their child vaccinated.	36.2% of parents were hesitant to get their child vaccinated, while 6.3% refused.	The major reason for vaccine hesitancy was the short time of vaccine development and fear of side effects.
19.	Al-Nafeesah, A.S. et al., 2021 [29]	Saudi Arabia	Mean age = 33 years College level 89%	Cross-sectional Online survey	1143 parents of children <6 years of age 88% mothers	Overall, the acceptance rate of parents was 36% regarding COVID-19 vaccination for their children.	38% of parents hesitated to vaccinate their child against COVID-19, while 26% parents refused.	The reason for hesitant behavior was the lack of information.
20.	Samannodi, M. et al., 2021 [30]	Saudi Arabia	Mean age = 37 years College level 85.4%	Cross-sectional Non-random sampling Online survey	508 parents 61.3% mothers	Overall, 69.3% parents presented acceptance to get their child vaccinated against COVID-19.	30.9% of parents were hesitant to get their children vaccinated.	The major reason for vaccine hesitancy was lack of information regarding vaccine effectiveness.
21.	Almusbah, Z. et al., 2021 [31]	Saudi Arabia	Mean age = Not given College level (no %)	Cross-sectional Non-random sampling Online survey	1000 parents 47% mothers	Among study subjects, 28.1% parents accepted vaccination for their children.	Overall, 37.3% of parents presented hesitant behavior. And 34.6% of parents rejected the vaccination.	The fear of side effects was the main reason for vaccine hesitancy.
22.	Kocamaz, E.B. et al., 2022 [32]	Turkey	Mean age = 38.9 years College level 82.3%	Cross-sectional Non-random sampling Online survey	384 parents of children <18 years of age 68.8% mothers	Overall, 79.2% parents gave acceptance to get their children vaccinated.	20.8% of parents presented hesitancy regarding getting their child vaccinated.	The parents were hesitant because of insufficient information regarding vaccine side effects and little evidence of vaccine safety.
23.	Alhazza, S.F. et al., 2021 [33]	Saudi Arabia	Mean age = 35 years College level 89.4%	Cross-sectional Online survey	1052 parents 51.2% mothers	63% parents accepted vaccine for their children	37% of parents were hesitant or unsure about vaccinating their children	The main reason was the novelty of vaccine as well as the fear of side effects.
24.	Shmueli, L., 2021 [34]	Israel	Mean age = 45 years College level 86%	Cross sectional Non-random sampling Online survey	1012 parents of children aged 5–11 years	Overall, 57% parents accepted the COVID-19 vaccine for their children.	43% of parents were hesitant regarding vaccinating their children against COVID-19.	The reasons for hesitancy were issues regarding the safety of vaccine, fear of side effects and concerns about clinical trials.
25.	Al-Qerem, W. et al., 2022 [35]	Jordan	Mean age = 38 years College level 72.9%	Cross sectional Non-random sampling Online survey	819 parents 70.9% mothers	Overall, 274 (30.2%) accepted the vaccine for their children.	69.8% of parents were either hesitant or refused to get their children vaccinated against COVID-19.	Lack of adequate information regarding the safety and effectiveness of vaccination.

## Data Availability

Data are available upon a reasonable request.
